# Retrospective analysis of the perioperative outcome in living donor kidney transplantation with multiple renal arteries: does accessory vessel ligation affect the outcome?

**DOI:** 10.1007/s00345-024-04883-9

**Published:** 2024-03-15

**Authors:** Jacob Schmidt, Robert Peters, Josef Mang, Bernhard Ralla, Diana Elena Moldovan, Julia Dagnæs-Hansen, Lutz Liefeldt, Klemens Budde, Markus Lerchbaumer, Frank Friedersdorff

**Affiliations:** 1 Department of Urology, Charité, Universitätsmedizin Berlin, Freie Universität Berlin and Humboldt- Universität Zu Berlin, Charitéplatz 1, 10117 Berlin, Germany; 2 Department of Urology, Copenhagen University Hospital, Copenhagen, Denmark; 3 Department of Nephrology and Intensive Care, Charité, Universitätsmedizin Berlin, Freie Universität Berlin and Humboldt- Universität Zu Berlin, Charitéplatz 1, 10117 Berlin, Germany; 4 Department of Radiology, Charité, Universitätsmedizin Berlin, Freie Universität Berlin and Humboldt- Universität Zu Berlin, Charitéplatz 1, 10117 Berlin, Germany; 5 Department of Urology, Evangelisches Krankenhaus Königin Elisabeth Herzberge, Berlin, Germany

**Keywords:** Living donor kidney transplantation, Multiple arteries, Outcome, Doppler sonography

## Abstract

**Purpose:**

Accurate surgical reconstruction of arterial vascular supply is a crucial part of living kidney transplantation (LDKT). The presence of multiple renal arteries (MRA) in grafts can be challenging. In the present study, we investigated the impact of ligation versus anastomosis of small accessory graft arteries on the perioperative outcome.

**Methods:**

Clinical and radiological outcomes of 51 patients with MRA out of a total of 308 patients who underwent LDKT with MRA between 2011 and 2020 were stratified in two groups and analyzed. In group 1 (20 patients), ligation of accessory arteries (ARAs) and group 2 (31 patients) anastomosis of ARAs was performed.

**Results:**

Significant differences were observed in the anastomosis-, surgery-, and warm ischemia time (WIT) in favor of group 1. Students *t*-test showed comparable serum creatinine levels of 2.33 (± 1.75) to 1.68 (± 0.83) mg/dL in group 1 and 2.63 (± 2.47) to 1.50 (± 0.41) mg/dL in group 2, were seen from 1 week to 1 year after transplant. No increased rates of Delayed graft function (DGF), primary transplant dysfunction and transplant rejection were seen, but graft loss and revision rates were slightly higher when the ARAs were ligated. Analysis of Doppler sonography revealed that segmental perfusion deficits tend to regenerate during the clinical course.

**Conclusion:**

Ligation of smaller accessory renal arteries may not affect the outcome of living kidney transplantation, except for a minor increase in the reoperation rate. Segmental perfusion deficits of the graft seem to regenerate in most cases as seen in Doppler sonography.

## Introduction

Anatomic workup prior living donor transplantation (LDKT) is a crucial part in the selection of the donor nephrectomy side to avoid surgical complications in donor and recipient. Kidneys and their vascular supply is predisposed to congenital anomalies such as accessory renal arteries (ARAs) [1]. ARAs are present in 20 to 30% of the population and can occur bilaterally in nearly 10% [2]. Most commonly ARAs arise from the abdominal aorta supplying the superior or inferior pole of the kidney (normally smaller diameter size) or the renal hilum.

Using modern imaging technics, ARAs are normally well documented for preparing surgery. Multiphase contrast-enhanced computed tomography remains the gold standard imaging for anatomical workup with high diagnostic performance [3, 4].

Since accurate reconstruction of arterial vascular supply is crucial in LDKT, surgery can be challenging by the presence of multiple renal arteries (MRA), which may affect the outcome. There is an ongoing discussion if MRA are a relative contraindication for LDKT since studies suggested an increased risk of vascular and urological complications [5–7]. Although increasing numbers of renal transplantations with MRA have been performed in recent years due to improved surgical techniques, the presence of MRA in LDKT is controversial issue [8]. It remains unclear which conditions of ARAs are mandatory for successful anastomosis or ligation avoiding ischemic areas. Studies indicate a poorer 1-year graft survival in patients with MRA compared to single artery kidneys, without a difference in late complications [9, 10].

Thus, we investigated the impact of ligating small accessory graft arteries on perioperative outcome of LDKT with MRA.

## Methods

### Study cohort and data acquisition

The study cohort consisted of 51 patients who underwent LDKT with MRA between January 2011 and December 2020. A total of 308 LDKT were performed during this period at our center. Clinical characteristics, operative and radiologic outcomes for recipients were analyzed in a post hoc analysis using data from a prospective registry for LDKT at our center, Charité, Universitätsmedizin Berlin Campus Mitte [11]. Patients were stratified into two groups: group 1 included ligation of ARAs, and group 2 contained grafts with two or more anastomosed arteries. The mean follow-up period was 45.35 (± 13.71) months in group 1 and 46.20 (± 20.61) months in group 2 (*p* = 0.86). Information on perioperative events and outcomes were obtained from surgical and medical reports as well as from TBase©, an integrated electronic health record and research database for kidney transplant recipients [12].

The study was conducted in accordance with the amended Declaration of Helsinki and the guidelines of the institutional review board “Ethikkomission Charité” (Berlin, Germany) which based on the ICH Guideline for Good Clinical Practice.

### Radiologic analysis

During the clinical course of the recipients, daily routine ultrasonography with B-mode, Doppler and resistance index measurements were performed for 14 days postoperatively. Diagnostic reports from our radiology information system were analyzed. CT imaging of donors showing vascular anatomy was performed preoperatively as a standard part of the transplant evaluation process on 16- to 320-slice CT scanners with 0.5–1-mm slice collimation or less. Iodine-containing contrast medium was intravenously applied as standard within the biphasic (arterial and venous phase) imaging protocol (Ultravist^®^, 370 mg iodine/ml, Bayer HealthCare Pharmaceuticals, Berlin, Germany; Xenetix^®^, 350 mg iodine/ml, Guerbet, Roissy, France). Images reconstructed with a slice thickness of 0.5–1 mm were used to analyze and measure the diameters of the ARA.

### Statistical analysis

Statistical analysis of the data was performed using Microsoft Office Excel 2022 and IBM SPSS Statistics 26 software. Student *t* test was used for continuous variables and chi-square test for nominal variables. *P* values < 0.05 were considered as statistically significant.

## Results

Of the 51 recipients of living donor grafts with MRA, anastomosis of ARAs to the blood system was performed in 31 cases (group 2). In this group, anastomosis of three renal arteries was performed in only one graft. In the remaining 20 recipients, the accessory vessel was ligated (group 1). As seen in Table 1, no significant difference between the two groups in age, sex, Body Mass Index (BMI) or days of hospitalization was observed. In addition, comparable serum creatinine levels of 2.33 (± 1.75) to 1.68 (± 0.83) mg/dL in group 1 and 2.63 (± 2.47) to 1.50 (± 0.41) mg/dL were seen from one week to one year after transplant. Significant differences between the two groups were shown in the operative time for anastomosis (group 1: 37.05 (± 7.04) min vs. group 2: 44.45 (± 10.46) min; *p* = 0.0008), surgery (group 1: 156.15 (± 46.86) min vs. group 2: 188 (± 41.89) min; *p* = 0.015) and warm ischemia time (WIT) with shorter durations in group 1 as shown in Table 1 (group 1: 127.9 (± 47.45) sec vs. group 2: 165.61 (± 41,88) sec; *p* = 0.004). Also, cold ischemia time was markable shorter in group 1.

**Table 1:** Patients and surgery specific characteristics: mean (± SD or percentage of group); *p-value < 0.05 in chi-square or students *t* test

Parameter	Ligature of accessory artery (*n* = 20) (group 1)	Anastomosis of accessory artery (*n* = 31) (group 2)	*p* value
Recipient age (years)	44.55 (± 15.32)	42 (± 16.42)	0.58
Gender			
Female	5 (25%)	7 (22.6%)	0.84
Male	15 (75%)	24 (77.4%)	0.84
Body Mass Index (kg/m^2^)	24.61 (± 3.73)	25.88 (± 3.90)	0.27
Hospital stay (days)	15 (± 6.20)	15.84 (± 7.20)	0.68
Follow-up (months)	45.35 (± 13.71)	46.20 (± 20.61)	0.86
Serum creatinine (mg/dl) post-surgery			
1 week	2.33 (± 1.75)	2.63 (± 2.47)	0.65
1 month	1.80 (± 0.67)	1.62 (± 0.58)	0.33
3 months	1.78 (± 0.67)	1.57 (± 0.44)	0.21
6 months	1.86 (± 1.01)	1.63 (± 0.45)	0.31
12 months	1.68 (± 0.83)	1.50 (± 0.41)	0.36
Operative time for Anastomosis (minutes)	37.05 (± 7.04)	44.45 (± 10.462)	*0.008
Duration of surgery (minutes)	156.15 (± 46.86)	188 (± 41.89)	*0.015
Cold ischemia time (minutes)	138.70 (± 37.99)	157.45 (± 33.37)	0.07
Warm ischemia time (seconds)	127.9 (± 47.45)	165.61 (± 41.88)	*0.004

The measured vessel diameter on CT of the ARA in group 1 ranged from 1.1 to 3.0 mm with a mean of 1.88 (± 0.57) mm, whereas diameter range in group 2 was 1.8 mm to 4.4 mm with a significant higher mean of 3.05 (0.75) mm (*p* < 0.001). In both groups, left living donor nephrectomies followed by organ transplantation into the right iliac fossa were predominant, accounting for nearly 90%. In group 1, the majority of ARAs were located at the superior pole of the kidney (75%) (*p* < 0.001). In contrast, 77.4% of the accessory vessels in group 2 were found at the inferior pole (*p* < 0.001) (Table 2).

**Table 2:** Vascular characteristics, graft perfusion and complication: diameter in millimeter (SD) or number (percentage of group), *p-value < 0.05 chi-square or students *t* test

Diameter of accessory arteries	Ligature of accessory artery (*n* = 20) (group 1)	Anastomosis of accessory artery (*n* = 31) (group 2)	*p* value
Minimum (mm)	1.1	1.8	
Maximum (mm)	3	4.4	
Mean (mm)	1.88 (± 0.57)	3.05 (0.75)	* < 0.001
Location of accessory arteries			
Inferior pole	4 (20%)	24 (77.4%)	* < 0.001
Organ middle	1 (5%)	5 (9.8%)	0.23
Superior pole	15 (75%)	2 (6.5%)	* < 0.001
Graft perfusion			
Intraoperative perfusion deficit described	8 (40%)	5 (16.1%)	0.05
Segmental perfusion deficit in postoperative Doppler sonography	3 (15%)	4 (12.9%)	0.83
Regeneration of perfusion deficit in Doppler sonography	3 (100%)	2 (50%)	0.15
Mean diameter of accessory arteries with perfusion deficit in Doppler sonography (mm)	1.87 (± 0.86)	2.75 (± 0.98)	0.79
Complication			
Graftloss	2 (10%)	2 (6.5%)	0.65
Delayed graft function	2 (10%)	3 (9.7%)	0.97
Primary transplant dysfunction	1 (5%)	2 (6.5%)	0.83
Transplant rejection	4 (20%)	6 (19.4%)	0.96
Intraoperative bleeding event	2 (10%)	4 (12.9%)	0.59
Revision (Clavien–Dindo IIIb) within 30 days	3 (15%)	2 (6.5%)	0.32
Ureteral necrosis with subsequent need for ureteroneocystostomy	1 (5%)	1 (3.2%)	0.75
Endourological operation	3 (15%)	2 (6.5%)	0.32
Transplant nephrectomy	2 (10%)	1 (3.2%)	0.31

**Fig. 1:**
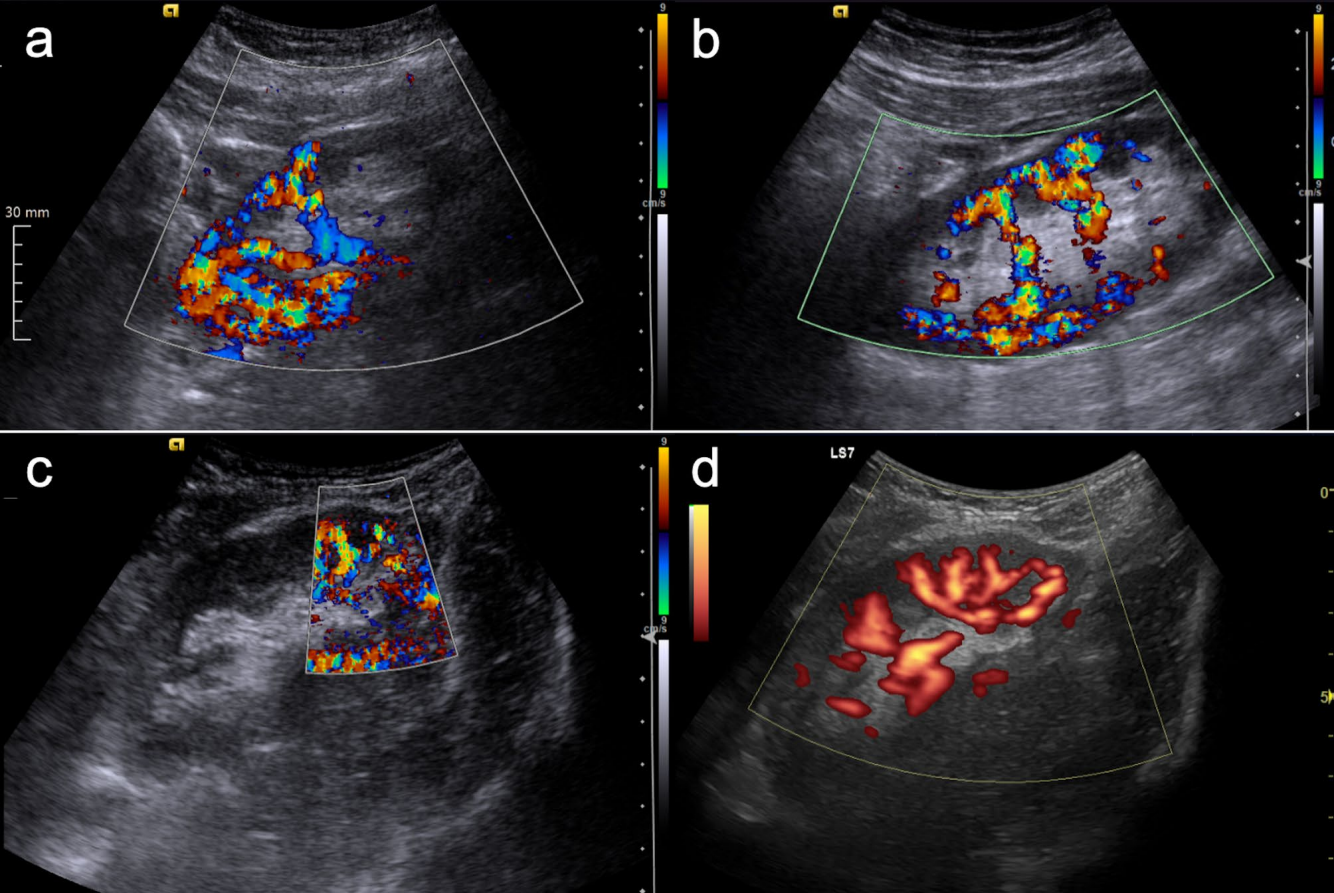
Doppler sonographic findings indicate regeneration of a segmental perfusion deficit during the clinical course in living donor kidney grafts with ligated accessory artery 2 (**a**), 4 (**b**), and 7 days after transplantation (**c**). No marked shrinkage of the parenchyma was observed after 3.5 years (**d**)

An intraoperatively macroscopically visible segmental perfusion deficit of the graft was described more frequently in group 1 with 40% than in group 2 with 16.1% (*p* = 0.05). Postoperative Doppler ultrasound revealed segmental perfusion failure in 3 (15%) grafts in group 1 and 4 (12.9%) grafts in group 2 (*p* = 0.83). Regeneration of renal perfusion deficit during the clinical course was 100% in group 1 and 50% in group 2, respectively (*p* = 0.15) (Table 2, Fig. 1). Mean diameter of the ligated vessel and following sonographic visible perfusion deficit was 1.87 mm (± 0.86) versus 2.75 mm (± 0.98) of the anastomosed ARA (*p* = 0.79).

Delayed graft function (DGF), defined as need for dialysis within one week after transplant, was observed in both groups almost in the same range of about 10% (*p* = 0.97) (Table 2). In addition, transplant rejection occurred in nearly 20% in both groups (*p* = 0.96), whereas complete primary graft dysfunction was seen slightly more often in group 2 and graftloss in group 1 (10% vs. 6.5%; *p* = 0.65). Intraoperative bleeding events were described in approximately the same range (10.0% and 12.9%; *p* = 0.59) in both groups.

A Clavien–Dindo IIIb complication, requiring surgical intervention under general anesthesia within 30 days, occurred more frequently in group 1 at 15% versus 6.5% in group 2 (*p* = 0.32) as seen in Table 2 [13]. Reasons for reoperation during this period included mainly fascia and wound reconstruction, lymphocele surgery in one case and ureteral stent dislocation leading to macrohematuria in another case. Ureteroneocystostomy (UCN) due to manifest ureteral necrosis had to be performed in one case 36 days after transplantation in group 1 and in one case in group 2 after 166 days (*p* = 0.75) with previous need insertion of a ureteral stent and nephrostomy due to hydronephrosis. Overall, the number of endourologic procedures such as insertion of a ureteral stent and nephrostomy due to hydronephrosis or ureterorenoscopy for the treatment of urolithiasis was higher in group 1 (15% versus 6.5%; *p* = 0.32). In group 1, transplant nephrectomy had to be performed in one case because of acute graft infection after renal puncture with consecutive hematoma 12 months after transplantation and in another case because of a prolonged history of recurrent urolithiasis and graft failure after 46 months. In group 2, only one transplant nephrectomy was performed after 181 days because of transplant failure and patient´s intolerance to immunosuppressants (10% vs. 3.2%, *p* = 0.31). Graftloss, as described above, has occurred for corresponding reasons and in one case due to a recurrent multidrug-resistant bacteria infection.

## Discussion

With the development of surgical expertise and minimally invasive techniques, as well as general organ scarcity, LDKT with MRA has become increasingly common in recent years [14]. The literature describes that MRA kidney transplantation may be associated with higher complication rates and DGF [7]. In this context, the effects of ligating accessory vessels in MRA grafts compared with anastomosis to the recipient's blood system are the subject of surgical concern. It is discussed that inadequate ligation of accessory arteries may lead to severe postoperative complications such as acute ureteral necrosis or renal infarction [15, 16]. On the other hand, prolonged operative times for anastomosing accessory branches and increased complexity of the operation may affect the outcome of short- and long-term postoperative graft function.

In the present study, we retrospectively investigated the impact of anastomosing versus not anastomosing accessory vessels in MRA-grafts on the postoperative outcome. In our cohort, significant differences between the two groups were seen in the operative time for the anastomosis, time of surgery and WIT, but not in creatinine levels up to 1 year after transplantation, DGF or primary transplant dysfunction and transplant rejection. Interestingly, the operative time was longer than the operative time for the anastomosis alone, suggesting that the complexity of the procedure extends beyond the vascular anastomosis in the presence of MRA. The prolonged operative time may be attributed to several factors, including deviations from standard surgical procedures, the challenge of maintaining precise graft bed positioning, and the need to avoid kink stenosis in the anastomosed arteries under a vigilant renal perfusion monitoring. In this context, detailed anatomical workup prior to LDKT with complex vascular anatomy, newer imaging techniques, such as three-dimensional visualization using virtual reality, could contribute to more detailed planning of the surgery and thus may help to shorten the operative time [17]. Thus, in contrast to the literature described correlation, no negative influence of prolonged operation times on graft function when ARAs were anastomosed was detected [18]. In a study in which the cohort of our center was analyzed from 2011 to 2016, Zeuschner et al*.* were able to determine a DGF rate of 6.3% [19]. In comparison, the present study showed a slightly higher DGF rate of 10% in the presence of MRA. Nevertheless, the incidence of DGF is still at a low level, consistent with existing literature and less than the comparison group of Zeuschner et al*.* with 11.5% (robot-assisted versus laparoscopic donor nephrectomy), so that a mere increase in DGF due to MRA is not proven [7].

Proposed by Iwami et al*.* a cut-off diameter of 2 mm seems worth attempting regarding the success rate and graft function, while cutting of a 2-mm ARA leads to parenchymal loss of less than 8% [20]. Although the mean diameters of ARAs in both groups differed from 1.88 mm to 3.05 mm, our study did not identify a clear cut-off value for performed anastomosis or ligation in our center. Intraoperative described perfusion deficits were seen more frequently in group 1, but in most cases, they could no longer be detected in standardized follow-up Doppler ultrasound during the early postoperative phase (2 weeks). Interestingly, postoperative Doppler sonography showed no significant higher rate of segmental perfusion deficit after ligation. Furthermore, regeneration of the perfusion deficit was seen in group 1 in all cases observed (Table 2, Fig. 1). An underlying mechanism for the complete regeneration may be the small average diameter of the ligated accessory branch, thus supplying a small graft segment that can be compensated by intraparenchymal vessels and a possible formation of collateral vessels could play a role. Ligation of larger accessory arteries would likely lead to necrosis without regeneration. Therefore, vascular events like arterial stenosis in the larger anastomosed ARAs with a wider graft supply area may lead to a lasting segmental infarction in some cases (in group 2). The exact mechanisms remain unclear due to the retrospective nature of the study.

Operative revision in the early postoperative period (Clavien–Dindo IIIb) appeared to be more frequent when ARAs were ligated (Table 2), but as mentioned above, the reasons for reoperation were not vascular, ureteral or bleeding complications, but mainly fascia or wound dehiscence and therefore association with the presence of MRA is unclear. Furthermore, transplant nephrectomies were performed more frequently in group 1, but again, no surgical or other reason related to the presence of MRA was seen.

Moreover, our results were concordant with literature describing the localization of the ARA as a further important parameter, since ligation of an inferior polar branch may reduce nutrient supply to the ureter and therefore cause ureteral necrosis [15]. Although most inferior polar vessels were anastomosed and equal numbers of ureteral necrosis were observed in both groups, the one manifest ureteral necrosis in group 1 was seen in the case of a ligated lower pole artery and also in group 2, the ARA was located at the lower pole. Furthermore, Kok et al*.* could show a significantly higher rate of urological complications after ligation of inferior pole arteries [21]. In the present study, endourologic procedures were also found to be performed more frequently in group 1, but only the ligation of superior pole arteries was done in the underlying cases. Because upper pole vessels do not usually cause ureteral affection, it remains unclear whether their ligation is the definitive reason for the higher rate of urologic complications in the present study [7].

Overall, the results of our study are consistent with the results of the existing literature identifying MRA grafts as those with a good long-term outcome with modestly increased complication rates and thus recommends them for transplant consideration [6, 8, 9, 14, 22]. When it comes to the question whether ARA ligation causes a higher complication rate, no statistical significance was found, but the rate of reoperations was slightly higher in the group with ligated vessels due to unspecific reasons.

Our study had several limitations. It is a retrospective observational study, which was performed at a single center and therefore its generalizability is limited. Furthermore, the number of patients was small, but comparable to literature. Although the demographic characteristics of the two groups were similar, differences in the localization and diameter of the ligated artery were observed. Therefore, the comparability between the two groups in this real-world data analysis is somewhat constrained. To address this topic in the future, these results should be confirmed in a prospective, multicenter study.

## Conclusion

This study indicates that ligating smaller accessory renal arteries, within the limits of careful surgical consideration and necessary caution, may not necessarily adversely affect the outcome of living kidney transplantation except for a minor increase in the overall reoperation rate. A possible segmental perfusion deficit of the graft can regenerate during the clinical course as seen in Doppler sonography.

## Data Availability

The data that support the findings of this study are available on request from the corresponding author.
